# HIV status among women with mental illness

**DOI:** 10.1186/1753-6561-5-S6-P328

**Published:** 2011-06-29

**Authors:** T Jeyaseelan Senthinath, T Senthilkumar, P Revathi

**Affiliations:** 1 Microbiology, Chennai Medical College Hospital & Research Centre, Trichy, India; 2 Anbalaya, Trichy, India; 3 Pharmacology, Chennai Medical College Hospital & Research Centre, Trichy, India

## Introduction / objectives

Mentally ill women (MIW) are sexually exploited. Despite, their HIV Status is less studied. Present report aims to study the HIV status of MIW and highlight the difficulties encountered while handling them.

## Methods

HIV status was screened by standard methods recommended by National AIDS Control Organization, INDIA for 50 young women with various mental illnesses. Difficulties encountered while handling them were elicited with the help of care takers. The data were analysed by simple descriptive statistics.

## Results

The age of the subjects varied from 19 - 34, with the median 25 yrs. They were wandering on the road & were sexually exploited for their basic needs. Cheap & easy availability, sexual perversion, substance abuse, Lack of family support, inability to stress condom usage to partners leads to dissemination of HIV in the society. HIV was positive (64%) among them & CD4 count was low as much as 14%. Highly Active Anti-Retroviral Therapy (HAART) could not be initiated due to non-acceptance, non-cooperation, running away from shelter/ rehabilitation home, non-compliance of drug intake, & issues related to underlying disease. They were neither aware of condom nor did they practice any protective measure.

## Conclusion

As handling MIW has multiple problems there is a need for 1 to 1 counselling to protect them, which will prevent the spread of HIV and initiate HAART therapy for them. The authorities and the public should be made aware of this mode of dissemination of HIV and measures should be taken to prevent them.

## Disclosure of interest

None declared.

